# Camptodactyly: From Embryological Basis to Surgical Treatment

**DOI:** 10.3390/medicina59050966

**Published:** 2023-05-17

**Authors:** Jan Kloc, Boris Dzula, Ivan Varga, Martin Klein, Boris Steno

**Affiliations:** 1 Department of Orthopaedic Surgery, Faculty Hospital of J.A. Reiman, Holleho 14, 080-01 Presov, Slovakia; kloc.j@fnsppresov.sk (J.K.); dzula@fnsppresov.sk (B.D.); 2 Institute of Histology and Embryology, Faculty of Medicine, Comenius University in Bratislava, Spitalska 24, 813-72 Bratislava, Slovakia; ivan.varga@fmed.uniba.sk; 3 II. Department of Orthopaedic and Trauma Surgery, Faculty of Medicine, Comenius University in Bratislava, Spitalska 24, 813-72 Bratislava, Slovakia; boris.steno@fmed.uniba.sk

**Keywords:** camptodactyly, flexor digitorum longus, proximal interphalangeal joint, surgical treatment

## Abstract

Camptodactyly is a relatively rare hand deformity presenting as the proximal interphalangeal joint’s nontraumatic and progressive flexion contracture. Most cases are limited to the fifth finger. The severity and type of camptodactyly should be considered to optimize treatment. Since many structures at the finger base can be involved in the pathogenesis of the deformity, surgical treatment for this particular type of deformity is challenging. This paper aims to bring insight into camptodactyly’s pathogenesis and treatment options. We discuss the indication and pitfalls of surgical treatment options for particular camptodactyly types and present a case of a fourteen-year-old boy who was admitted to our department with proximal interphalangeal joint flexion contracture of the left fifth digit.

## 1. Introduction

Camptodactyly is a nontraumatic and progressive flexion deformity that most commonly affects the proximal interphalangeal joint (PIPJ) of the fifth finger and may or may not include the other fingers, while the first finger is always spared [1,2,3,4]. It affects approximately less than 1% of the population [2]. Around two-thirds of cases are present bilaterally, although the degree of contracture is not symmetrical [4]. The deformity generally increases during a growth spurt from one to four years of age and from ten to fourteen [3,5,6].

The primary cause of camptodactyly is still discussed [7,8,9]. Abnormalities in all structures that cross the proximal interphalangeal joint are present in camptodactyly [9]. The most common pathologies include tightness or shortness of flexor digitorum superficialis, abnormal lumbrical origin and insertion, and volar skin deficits. These changes result in flexion of the PIPJ. Due to the contracture of PIPJ, secondary changes develop, such as adhesions of the dorsal apparatus and lateral bands, a deficient dorsal central slip extensor mechanism, volar plate contracture, and tightness of the collateral ligaments [1,9,10,11]. The long-term deformity can lead to bone changes in the proximal and middle phalanges and the joint surfaces of PIPJ [12,13].

## 2. Classification

From a clinical point of view, Siegert et al. [3] divided camptodactyly into simple and complex types. In simple type cases, the flexion contracture affects only the proximal interphalangeal joint. Other associated deformities exist in complex cases, such as syndactyly or a combination of clinodactyly and camptodactyly. Glicenstein et al. [5] distinguish primary and secondary camptodactyly. Primitive camptodactyly appears in the first years of life. It affects both sexes equally and progresses with skeletal growth. It may also appear close to adolescence, with predominance in females. Secondary camptodactyly is associated with syndromes and other malformations and typically involves more than one finger. The most frequently associated pathologies are radial club hand, arthrogryposis, Marfan syndrome, and oculodentodigital syndrome. Finally, Benson et al. [6] classified camptodactyly into three types: infantile, adolescent, and syndromic:

Type I (infantile) is the most typical form. The little finger is most often affected, and cosmetic complaints are more common than functional impairments.

Type II (adolescent) occurs predominantly in females between the ages of seven to eleven years. Clinically, it resembles type I, with subtle initiation and progressive evolvement.

Type III (syndromic) camptodactyly is present from the time of birth. It usually affects multiple fingers; bilateral deformities with severe fixed contractures are constant. Syndromic camptodactyly causes more discomfort than infantile and adolescent types.

## 3. Embryological Background

From the embryological perspective, limb development is a highly complex and tightly orchestrated process which starts around Day 26 (4th week) after fertilization as limb buds *(gemmae membrorum)* and is finished by the 8th week. The upper limb emerges as an outgrowth of the somatic lateral plate mesoderm and somatic mesoderm between the 9th and 10th somite in the presumptive upper limb-forming field [14]. The growing mesoderm elevates the overlying ectoderm forming the apical ectodermal ridge *(crista ectodermalis apicalis)*, essential for limb development in the proximo-distal axis. The limb patterning occurs in two other axes, namely the anteroposterior originating from the zone of polarizing activity and the dorsoventral axis. Each depends on a specific gene expression sequence. The proximo–distal, anterior–posterior, and dorso–ventral axes are predominantly controlled by the fibroblast growth factor, sonic hedgehog, and Wnt signaling pathway, respectively [15,16]. The upper limb bud is subdivided into three zones giving rise to stylopod (future humerus), zeugopod (future radius and ulna), and autopod (future hand) developing in a proximo–distal sequence (i.e., the humerus forms first). Cells in each zone also have a unique gene expression pattern (autopod cells express HOXA13-HOXA 10-13) [17]. The congenital type of camptodactyly is inherited in an autosomal dominant manner. Along with syndactyly, camptodactyly is characterized as a soft tissue anomaly belonging to the category of handplate *(lamina manus)* formation/differentiation failures in an unspecified axis. The exact mechanisms behind dorsoventral and digit-specific ligament/tendon formation have been long under-researched. A gene encoding proteoglycan 4 (*PRG4* gene) has been thought of as the candidate gene responsible for this flexion-contraction disorder, but a more in-depth analysis providing deeper insights into the pathogenesis of this condition has been lacking [18]. In recent years, more light has been shed on the *PRG4* gene mutations, leading to the development of camptodactyly-arthropathy-coxa vara-pericarditis (CACP) syndrome. In the most recent 2023 paper, Bağrul et al. [19] corroborated previous reports that *PRG4* mutation can be behind the condition. The pathogenesis of CACP syndrome has been linked to the defective *PRG4* product called lubricin, found in the synovial fluid, cartilage surface, and tendons, which, besides its lubricating function, is also responsible for cell growth regulation. To make the issue even more complicated, some authors estimate that camptodactyly occurs as a feature within more than 50 different conditions [20].

## 4. Treatment Options

Historically, conservative treatment has been the first choice of camptodactyly management [6,21,22,23]. Non-surgical treatment involves the use of passive or dynamic splints and hand therapy. Benson et al. [6] suggest wearing static splints for 15–18 h daily. Hori et al. [22] prefer the usage of a dynamic splint for 24 h a day for the first months, followed by using splints for 8 h a day. Rhee et al. [23] propose a stretching protocol consisting of 5 min of passive stretching 20 times a day until the contracture is corrected and additional exercises 5 to 10 times daily. However, it has been shown that contracture tends to return after discontinuation of wearing the splint [21].

It is generally accepted that surgery should be reserved for failed cases of nonoperative management, where a fixed 60-degree flexion contracture of PIPJ has been reached [2,3,4,9,24,25]. The surgical procedures can be divided into those that identify and address the primary cause; those that try to rebalance the PIPJ through transferring flexion force to the extensor surface; those that provide the release of all structures of the volar face to achieve correction; and bone procedures with dorsal-angle osteotomy of the neck of the proximal phalanx [24]. The risk of flexion loss in the interphalangeal joint and limited extension improvement are essential factors when deciding whether to take a surgical approach to treatment [3]. Postoperative care, rehabilitation, and patient cooperation are crucial for a favorable outcome of surgery [11,24,25].

## 5. Case Report

**Figure 1:**
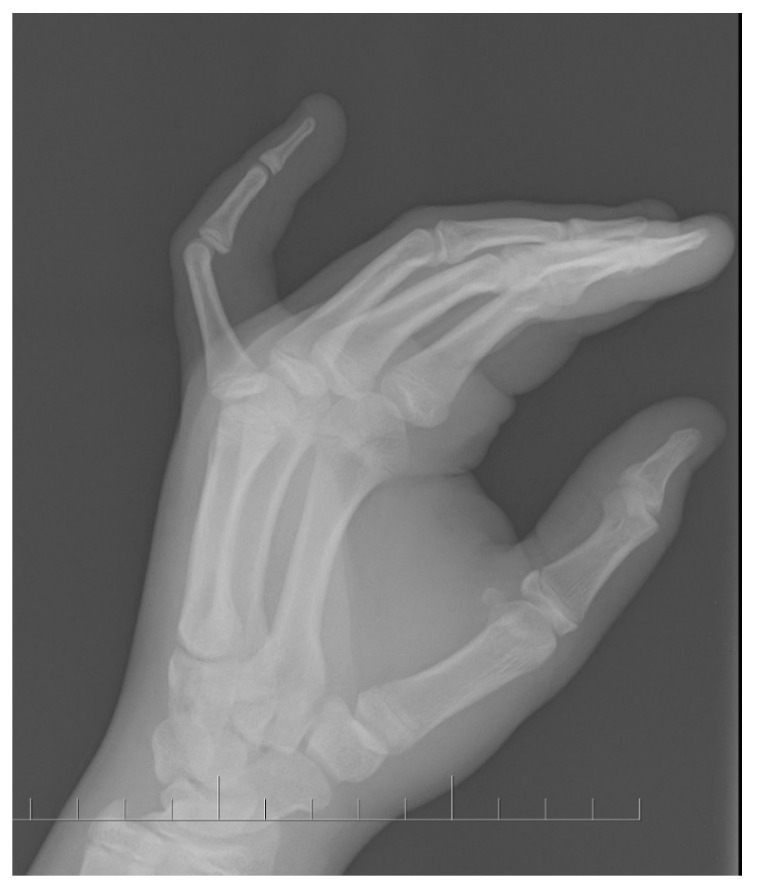
Lateral X-ray of the left hand with 60-degree PIPJ contracture.

A fourteen-year-old boy was admitted to the outpatient clinic with PIPJ flexion contracture of the left fifth digit. There was no history of hand injury. The X-ray showed 60° flexion PIPJ contracture without bone changes (Figure 1).

Clinically, the 60° extension deficit of PIPJ was nonreducible during passive manipulation. There was no deficit in PIPJ flexion, distal interphalangeal, and metacarpophalangeal joint motion. No sign of other hand deformity was present. The diagnosis of the established, rigid form of simple adolescent camptodactyly was set. Due to the extent of the deformity and unsuccessful nonoperative management, our patient was indicated for surgical treatment.

**Figure 2:**
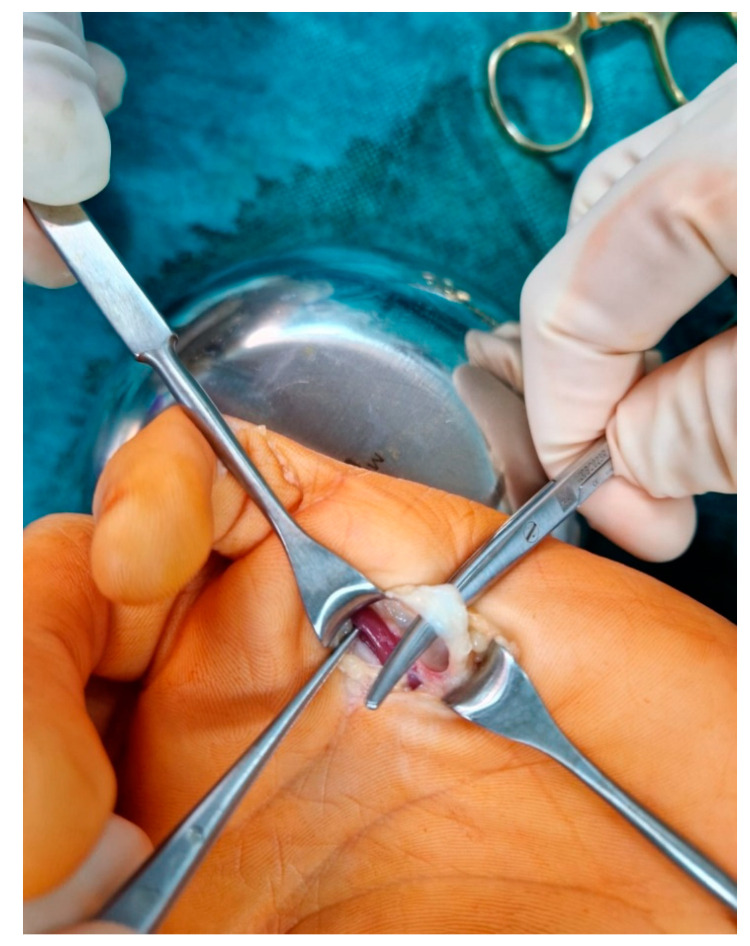
Transverse incision at the distal palmar crease, and the FDS released as distally as possible.

**Figure 3:**
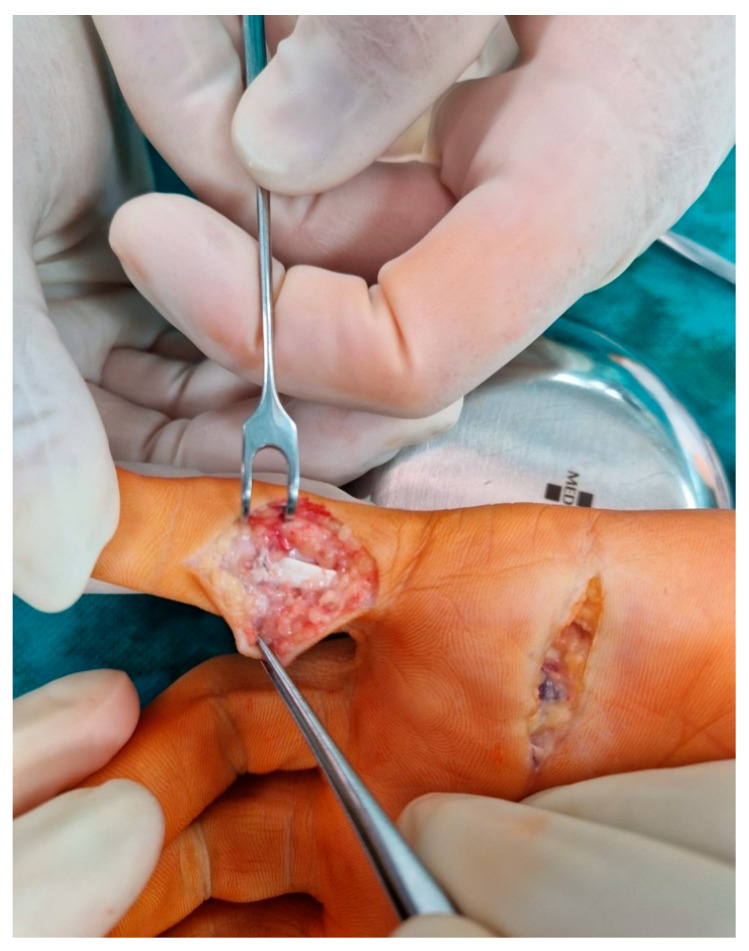
Second skin incision distally in the PIP region to release the lateral bands, lumbrical insertion, and FDS at the level of the chiasm.

A transverse incision was made in the distal palmar crease. The flexor digitorum superficialis (FDS) was located in low-quality tissue and released as distal as possible by flexing the digit (Figure 2). Because the contracture correction was only partial, the second skin incision was made distally in the PIP region in Bruner fashion to release the FDS at the level of the chiasm (Figure 3).

**Figure 4:**
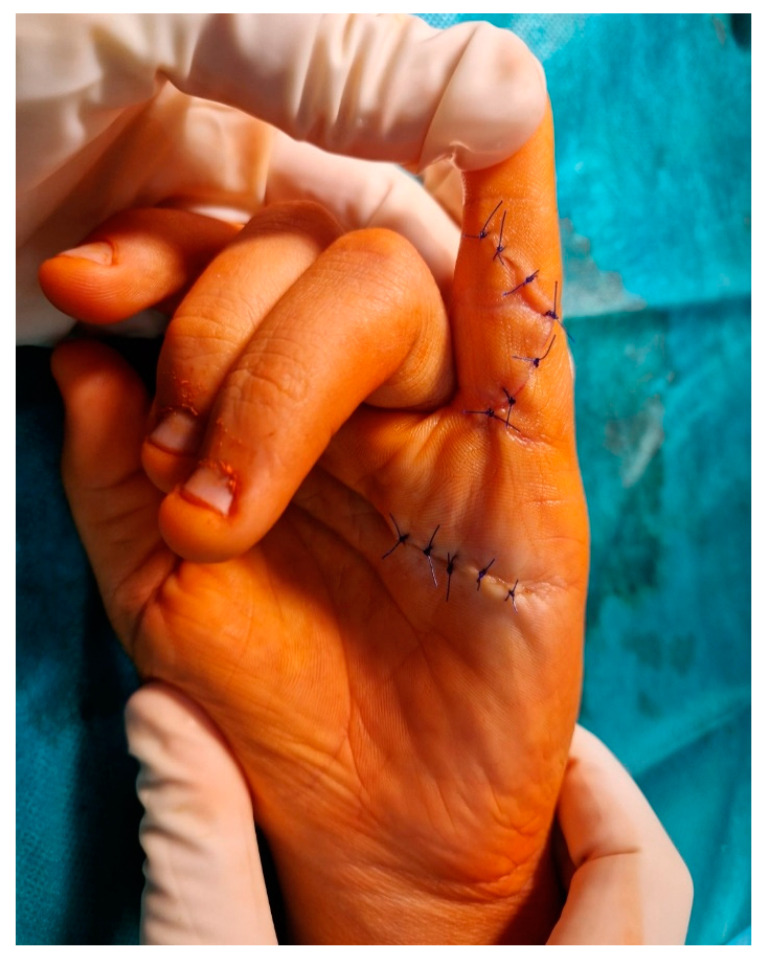
The final intra-operative result after correction of contracture.

**Figure 5:**
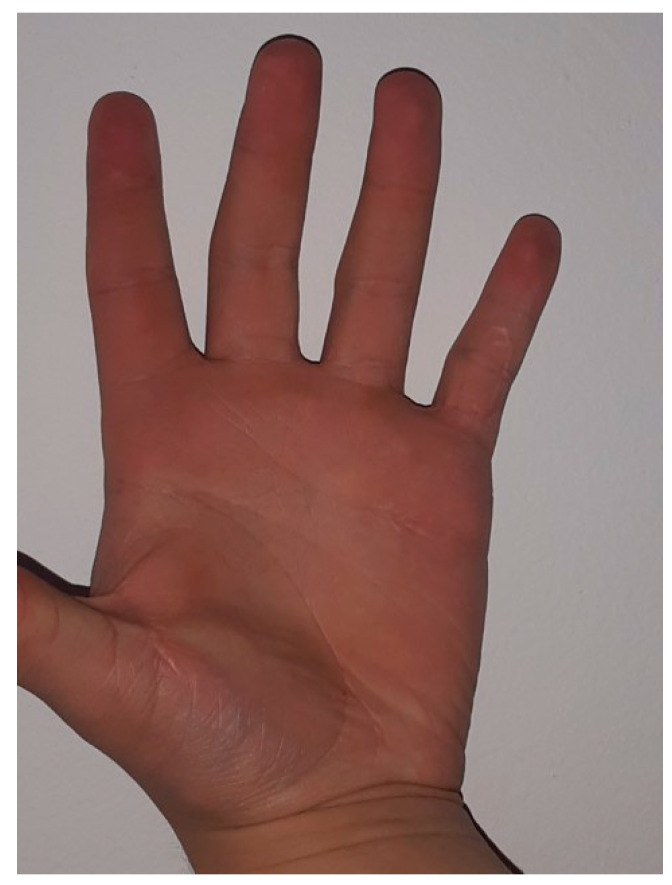
Nine months after surgery, full correction of deformity was achieved.

The aim was to release any tethered structures, lateral bands, and lumbrical insertion. After releasing all structures, a full extension was reached by gentle passive manipulation (Figure 4). The skin was sutured with 4–0 interrupted stitches. Then the volar cast, in the position of safety and ensuring full extension, was applied across the PIPJ for four weeks. Dressings were reduced and changed at two weeks. There was no evidence of central slip attenuation at four weeks, so active flexion was encouraged from four weeks, with night-time splintage utilized for six months post-operatively. Hand therapy was indicated during the first three months post-operatively. During follow-up, nine months after surgery, full deformity correction was observed (Figure 5) without extension and flexion PIPJ deficit.

## 6. Discussion

The pathogenesis of camptodactyly is not clear [7,8,9]. Although some cases occur sporadically, it has been proven to show an autosomal inheritance pattern [7,8]. Malik et al. [7] described a case of a German family with thirteen camptodactyly cases in four generations. Couser et al. have shown the association between the camptodactyly and deletion of the 22q11.21 chromosome [8].

The problem with camptodactyly management is that several types and forms of presentation exist, which means there is no single model for effective treatment. Generally, flexion deformities of less than 30 degrees do not interfere with daily life. On the other hand, more than 60 degrees contractures diminish function and require surgical treatment. Almeida et al. [24], in their retrospective assessment of 23 patients and a total of 40 fingers, observed that the cases of camptodactyly of the little finger alone in the flexible form (>60°) that underwent surgical treatment all presented excellent results. In the rigid forms, their observations indicated that there were benefits comprising gains of extension and correction of the deformity. However, the range of motion with active flexion of the proximal interphalangeal joint was always partial. Even in the cases with excellent results, there was an average loss of flexion of 15°. Over time, some cases evolved to present some loss of the gain previously achieved. The authors emphasized the need for continual follow-up monitoring, with systematic use of braces, until the final phase of skeletal growth [24].

The approach to deformities between 30 to 60 degrees differs. Many studies have demonstrated success with conservative management [6,21,22,23]. Unfortunately, there is no consensus on the most effective protocol, and the risk of recurrence remains high [21]. On the contrary, some authors recommend early surgical intervention [9,25]. Smith and Grobbelaar have introduced a unifying theory and approach to the surgical treatment of camptodactyly and demonstrated that with appropriate surgical technique, good to excellent results are achievable in 83% of patients according to the Siegert grading system [9]. They proposed that early palmar surgical release of the FDS in young children, with gentle passive manipulation to mobilize periarticular adhesions, may avoid the establishment of firmly fixed contractures and prevent secondary changes, which are more challenging to treat at higher age [9]. Based on this approach, Miranda et al. [11] described surgical treatment for established adolescent cases and presented well to excellent postoperative outcomes according to the Siegert grade in 87,5% of digits. One has to remember that excessive lengthening or tenotomy of the FDS decreases the active arc of motion and leads to loss of flexion, even if the procedure can provide a better extension of the PIPJ [25]. An incomplete extension is better tolerated than deficient flexion. Early mobilization after surgery should be instituted to promote flexion restoration [9,11,24].

In our fixed, established adolescent camptodactyly case, a simple release of FDS was not enough to reach full contracture correction. A full extension was achieved after the abnormal lumbrical insertion, and the lateral bands were released, followed by gentle passive manipulation. Although we consider the pathology of FDS as the most common in camptodactyly pathogenesis, addressing any other abnormalities is crucial. These include abnormal lumbrical origin and insertion, volar skin deficits, secondary changes, such as adhesions of the dorsal apparatus and lateral bands, a deficient dorsal central slip extensor mechanism, volar plate contracture, and tightness of the collateral ligaments. This notion agrees with the findings of the retrospective multi-centre study of 59 surgically treated patients presented by Corain et al. [26]. They concluded that Malek’s cutaneous approach and stepwise release of the retracting soft tissues allow prompt evaluation of the anatomical structures involved in the deformity and seem to be an effective surgical correction in the long term [26].

In cases of syndromic camptodactyly, a distinctive view of the surgical treatment occurs. A one-stage extension shortening osteotomy of the proximal phalanx has shown promising results. The Korean authors considered the extension osteotomy a straightforward and effective technique for improving finger function through the indirect lengthening of volar structures without the flexor tendon lengthening. The authors highlighted the suitability of this simple procedure for surgery on multiple fingers in patients with syndromic camptodactyly [13].

## 7. Conclusions

Camptodactyly remains a puzzling condition. Although its embryological background and exact etiopathogenesis are poorly understood, the conservative and surgical approaches yield favorable results. Our case report demonstrated that a full correction could be achieved when an appropriate surgical procedure is chosen. However, the main pitfall is the heterogeneity of its presentation, preventing the routine application of a single best surgical model.

## Data Availability

The data that were presented in this study are available on request from the corresponding author. The data are not publicly available due to privacy restrictions.

## References

[1] Smith R.J., Kaplan E.B. (1968). Camptodactyly and simialr atraumatic flexion deformities of the proximal interphalangeal joints of the fingers. A study of thirty-one cases. J. Bone Jt. Surg. Am..

[2] Jones K.G., Marmor L., Lankford L.L. (1974). An Overview on New Procedures in Surgery of the Hand. Clin. Orthop. Relat. Res..

[3] Siegert J.J., Cooney W.P., Dobyns J.H. (1990). Management of simple camptodactyly. J. Hand Surg. Br..

[4] Hamilton K.L., Netscher D.T. (2015). Evaluation of a stepwise surgical approach to camptodactyly. Plast. Reconstr. Surg..

[5] Glicenstein J., Haddad R., Guero S. (1995). Surgical treatment of camptodactyly. Ann. Chir. Main Memb. Superiour.

[6] Benson L.S., Waters P.M., Kamil N.I., Simmons B.P., Upton J. (1994). Camptodactyly: Classification and results of nonoperative treatment. J. Pediatr. Orthop..

[7] Malik S., Schott J., Schiller J., Junge A., Baum E., Koch M.C. (2008). Fifth finger camptodactyly maps to chromosome 3q11.2-q13.12 in a large German kindred. Eur. J. Hum. Genet..

[8] Couser N.L., Pande C.K., Walsh J.M., Tepperberg J., Aylsworth A.S. (2017). Camptodactyly and the 22q11.2 deletion syndrome. Am. J. Med. Genet. A.

[9] Smith P.J., Grobbelaar A.O. (1998). Camptodactyly: A unifying theory and approach to surgical treatment. J. Hand Surg. Am..

[10] McFarlane R.M., Classen D.A., Porte A.M., Botz J.S. (1992). The anatomy and treatment of camptodactyly of the small finger. J. Hand Surg. Am..

[11] Miranda B.H., Talwar C., Horwitz M.D., Smith P.J. (2022). Aggressive paediatric camptodactyly: The evolution of a proposed treatment algorithm. J. Plast. Reconstr. Aesthet. Surg..

[12] Netscher D.T., Hamilton K.L., Paz L. (2015). Soft-Tissue Surgery for Camptodactyly Corrects Skeletal Changes. Plast. Reconstr. Surg..

[13] Park B.K., Kim H.W., Park H., Park M.J., Hong K.B., Park K.B. (2020). One-Stage Extension Shortening Osteotomy for Syndromic Camptodactyly. J. Clin. Med..

[14] Guéro S. (2018). Developmental biology of the upper limb. Hand Surg. Rehabil..

[15] Al-Qattan M.M., Kozin S.H. (2013). Update on embryology of the upper limb. J. Hand Surg. Am..

[16] Dy C.J., Swarup I., Daluiski A. (2014). Embryology, diagnosis, and evaluation of congenital hand anomalies. Curr. Rev. Musculoskelet. Med..

[17] Montavon T., Le Garrec J.F., Kerszberg M., Duboule D. (2008). Modeling Hox gene regulation in digits: Reverse collinearity and the molecular origin of thumbness. Genes Dev..

[18] Oberg K.C., Feenstra J.M., Manske P.R., Tonkin M.A. (2010). Developmental biology and classification of congenital anomalies of the hand and upper extremity. J. Hand Surg. Am..

[19] Bağrul İ., Ceylaner S., Yildiz Y.T., Tuncez S., Aydin E.A., Bağlan E., Ozdel S., Bülbül M. (2023). A novel mutation in the proteoglycan 4 gene causing CACP syndrome: Two sisters report. Pediatr. Rheumatol. Online J..

[20] Krakow D., Rimoin D., Pyeritz R., Korf B. (2013). The Dysostoses. Emery and Rimoin’s Principles and Practice of Medical Genetics.

[21] Lethbridge K., Wollin L. (2014). A review of conservative management of camptodactyly in children and adolescents. Hand Ther..

[22] Hori M., Nakamura R., Inoue G., Imamura T., Horii E., Tanaka Y., Miura T. (1987). Nonoperative treatment of camptodactyly. J. Hand Surg. Am..

[23] Rhee S.H., Oh W.S., Lee H.J., Roh Y.H., Lee J.O., Baek G.H. (2010). Effect of passive stretching on simple camptodactyly in children younger than three years of age. J. Hand Surg. Am..

[24] Almeida S.F., Monteiro A.V., Lanes R.C. (2014). Evaluation of treatment for camptodactyly: Retrospective analysis on 40 fingers. Rev. Bras. Ortop..

[25] Evans B.T., Waters P.M., Bae D.S. (2017). Early Results of Surgical Management of Camptodactyly. J. Pediatr. Orthop..

[26] Corain M., Lando M., Pantaleoni F., Pozza P., Giardini M., Adani R. (2022). Surgical Treatment of Camptodactyly with Malek Cutaneous Approach and Stepwise Release: A Retrospective Multi-centre Study. J. Hand Surg. Asian Pac. Vol..

